# Isolation and characterization of phosphofungi, and screening of their plant growth-promoting activities

**DOI:** 10.1186/s13568-018-0593-4

**Published:** 2018-04-20

**Authors:** Xiaohui Wang, Changdong Wang, Junkang Sui, Zhaoyang Liu, Qian Li, Chao Ji, Xin Song, Yurong Hu, Changqian Wang, Rongbo Sa, Jiamiao Zhang, Jianfeng Du, Xunli Liu

**Affiliations:** 1 College of Life Science, Shandong Agriculture University, No. 61, Daizong Street, Taian, Shandong China; 2 College of Forestry, Shandong Agricultural Universities, No. 61, Daizong Street, Taian, 271018 Shandong China

**Keywords:** *Aspergillus niger*, Growth-promoting ability, HPLC, Illumina MiSeq sequencing, Phosphofungi

## Abstract

Rhizospheric microorganisms can increase phosphorus availability in the soil. In this regard, the ability of phosphofungi to dissolve insoluble phosphorus compounds is greater than that of phosphate-solubilizing bacteria. The aim of the current study was to identify efficient phosphofungi that could be developed as commercial microbial agents. Among several phosphate-solubilizing fungal isolates screened, strain CS-1 showed the highest phosphorus-solubilization ability. Based on phylogenetic analysis of the internal transcribed spacer region sequence, it was identified as *Aspergillus niger*. High-performance liquid chromatography analysis revealed that the mechanism of phosphorus solubilization by CS-1 involved the synthesis and secretion of organic acids, mainly oxalic, tartaric, and citric acids. Furthermore, strain CS-1 exhibited other growth-promoting abilities, including efficient potassium release and degradation of crop straw cellulose. These properties help to returning crop residues to the soil, thereby increasing nutrient availability and sustaining organic matter concentration therein. A pot experiment revealed that CS-1 apparently increased the assessed biometric parameters of wheat seedlings, implying the potential of this strain to be developed as a commercial microbial agent. We used Illumina MiSeq sequencing to investigate the microbial community composition in the rhizosphere of uninoculated wheat plants and wheat plants inoculated with the CS-1 strain to obtain insight into the effect of the CS-1 strain inoculation. The data clearly demonstrated that CS-1 significantly reduced the content of pathogenic fungi, including *Gibberella*, *Fusarium*, *Monographella*, *Bipolaris*, and *Volutella*, which cause soil-borne diseases in various crops. Strain CS-1 may hence be developed into a microbial agent for plant growth improvement.

**Electronic supplementary material:**

The online version of this article (10.1186/s13568-018-0593-4) contains supplementary material, which is available to authorized users.

## Introduction

Phosphorus is the second most important limiting element required for plant growth (Chai et al. 2011; Li et al. 2015a; Ram et al. 2015), and it cannot be substituted by any other nutrient. However, even though soils may contain a substantial reserve of total phosphorus (Collavino et al. 2010), most natural soils are typically deficient in this element. This is particularly true for highly weathered soils, in which phosphorus forms insoluble complexes with aluminum, iron, and hydroxides (in acidic soils), and with calcium (in alkaline soils) (Mendez 2014). To sustain crop production, the traditional method of rectifying phosphorus deficiency involved applying large amounts of phosphate fertilizers to the soil. However, only a small fraction of this added phosphate is available to plants, whereas a considerable proportion becomes immobilized after application (Singh 2011). This not only increases production costs but also leads to environmental pollution.

An alternative strategy involves a direct application of locally available rock phosphate (RP) to the soil; however, the effectiveness of RP as a fertilizer is highly dependent on the soil type, with the pH playing a particularly important role (Sulbarán et al. 2009). In this regard, Nahas (1996) reported a high correlation between the final pH and soluble phosphate content, with a decrease in pH directly influencing the solubilization of RP. Furthermore, RP is a non-renewable resource, and as such, its use as a fertilizer is not fully in accord with the current principles of sustainability (Ram et al. 2015).

It is well known that a number of bacteria and fungi are able to solubilize elemental phosphorus from insoluble RP for plant growth, and that fungi have a more pronounced ability to dissolve insoluble phosphorus compounds and have more stable genetic traits than those of bacteria (Gyaneshwar et al. 1998; Joseph and Jisha 2009; Rmn 1983). Collectively, such bacteria and fungi are referred to as phosphate-solubilizing microorganisms (PSMs), and include genera such as *Pseudomonas*, *Bacillus*, and *Rhizobium* (bacteria), and *Aspergillus* and *Penicillium* (fungi). Phosphorus-solubilizing activity is considered to be the most important among the multiple properties of the soil microorganisms that promote plant growth and nutrient absorption (Rodríguez and Fraga 1999). However, the growth-promoting effects of these PSMs are also influenced by environmental factors, including the compositional characteristics, organic matter content, texture, moisture, and pH of the soil, as well as the activities of certain enzymes (Ponmurugan and Gopi 2006). In addition, when introduced into the environment, PSMs should be able to compete with other soil microflora and successfully colonize the rhizosphere of crops. In this regard, there is a substantial advantage in using natural soil isolates as potential inoculants and in the same area from which they were isolated.

Thus far, research on PSMs has been insufficient, and few such organisms have been considered for exploitation as microbial fertilizer strains (Collavino et al. 2010; Yin et al. 2015). Given these considerations, the aim of the current study was to identify efficient phosphofungi from wheat rhizosphere soil that have the potential to be developed as commercial microbial agents.

## Materials and methods

### Soil sampling

The study site was a wheat field located in Taian City, Shandong Province, China. Wheat seedlings at the grain-filling stage were uprooted with intact roots and adhering soil from various sampling points. The aerial parts were excised using a sterilized knife, and the residual root portions were transferred to sterilized plastic bags (12 × 24 cm), which were labeled, sealed, placed in an icebox, immediately transported to the laboratory, and stored at 4 °C until use in subsequent experiments.

### Isolation and screening of phosphofungi

Non-rhizosphere soil was removed by gentle shaking, leaving behind only the rhizosphere soil. The rhizosphere soil was collected from roots by dipping and gentle shaking in sterile distilled water under aseptic conditions, and mixed on a table concentrator for 30 min. The resultant soil solution was then serially diluted up to 10^−6^-fold, and the respective dilutions were plated on Martin agar medium supplemented with 30 μg/mL streptomycin, and incubated at 28 ± 2 °C for 2–3 days (Bonito et al. 2016; Mehta and Nautiyal 2001). Colonies showing the predominant morphology were selected at random from each plate; isolates showing various colony morphologies were purified and screened for their phosphate-solubilizing ability. Overall, 20 colonies were incubated for 5–7 days on the National Botanical Research Institute phosphate growth (NBRIP) medium (Nautiyal et al. 2000). Halo formation around the colonies was considered to be indicative of phosphate-solubilization activity (Mehta and Nautiyal 2001).

### Phosphate-solubilization efficiency in liquid medium

Quantitative estimation of the phosphate-solubilization ability of the fungal isolates in broth was carried out using 50 mL of the NBRIP growth medium in 250-mL conical flasks inoculated with 1 mL of spore suspensions [inoculum concentration was with approximately 2–6 × 10^8^ colony-forming units (CFUs)/mL]. The NBRIP growth medium contained as follows (per 1 L): Ca_3_(PO_4_)_2_, 5.0 g; glucose, 10 g; (NH_4_)_2_SO_4_, 0.5 g; NaCl, 0.3 g; KCl, 0.3 g; MgSO_4_·7H_2_O, 0.3 g; FeSO_4_, 0.03 g; and MnSO_4_, 1.0 g, natural pH (5.51). The uninoculated medium served as a control. The inoculated medium was incubated at 30 °C and 200 r/min in the dark for 5 days. The entire experiment was performed in triplicate. Cultures were harvested by centrifugation at 1073×*g* for 10 min, and the supernatant was used in quantitative analysis. The amount of soluble phosphorus in the culture supernatant was estimated using the molybdenum blue method (Bray 1945). The results are expressed as mean values with standard deviations.

### DNA extraction, PCR amplification, and phylogenetic analysis

Mycelia were harvested after 4 days of growth on potato dextrose agar medium at 28 ± 2 °C and genomic DNA was extracted using an HP Fungal DNA kit (TransGen Biotech, Beijing, China), by following the manufacturer’s protocol. rDNA internal transcribed spacer (ITS) fragments were amplified using the universal fungal primers ITS1 and ITS4 (Pitkäranta et al. 2008). The obtained amplicons were sequenced by a commercial sequencing company (Sangon Biotech, Shanghai, China). The obtained nucleotide sequences were analyzed and compared with the ones deposited in the GenBank database using BLAST (https://blast.ncbi.nlm.nih.gov/Blast.cgi). A phylogenetic tree was constructed based on the neighbor-joining method. Genetic distances and similarity between the selected strains were computed using the maximum composite likelihood algorithm. The phylogenetic tree was constructed using MEGA 5.1 software (Mendez 2014), and the reliability of the tree topology was evaluated by bootstrap analysis with 1000 replicates.

### Dynamics of the phosphorus-solubilization mechanism of PSMs

The spore suspension (an inoculum containing approximately 2–6 × 10^8^ CFUs/mL) of the selected phosphate-solubilizing fungal (PSF) isolate was incubated in 50 mL of the NBRIP growth medium at 30 °C and 200 r/min in the dark for several days. An uninoculated medium served as a control. The culture supernatant was harvested by centrifugation at 1073×*g* for 10 min, daily, and then passed through a 0.22-μm sterile filter membrane. The pH, low-molecular-weight organic acids, and inorganic phosphate content of the supernatant were determined (Singh 2011). Fungal cell pellets were collected and dried in an oven at 70 °C to a constant weight, to determine the dry weight of the mycelium biomass (Mendes et al. 2013). The entire experiment was performed in triplicate, and the data are expressed as mean values with standard deviations.

Qualitative and quantitative analyses of organic acids in the culture supernatants were performed using an Agilent ZORBAX SB-Aq high-performance liquid chromatography (HPLC) system (Agilent Technologies, Inc., Santa Clara, CA, USA). Thermo C18 (250 mm × 4.6 mm, 5 μm) column was used (Agilent Technologies, Inc.). The mobile phase was 50 mM KH_2_PO4 (pH 2.70) and CH_3_OH (97:3); total run time was 30 min, with a flow rate of 0.8 mL/min; the injection volume was 2 μL; and the detection wavelength was 210 nm, with the analysis performed at 25 °C.

### Potassium-solubilization efficiency

Based on the similarity mechanisms of phosphorus and potassium-solubilization mechanisms, quantitative estimation of potassium-solubilization efficiency was conducted in silicate fermentation broth containing the following ingredients (per 1 L): sucrose, 10.0 g; (NH_4_)_2_SO_4_, 0.2 g; MgSO_4_·7H_2_O, 0.5 g; NaCl, 0.1 g; CaCO_3_, 0.1 g; and feldspar powder, 5.0 g, pH 7.0 ± 0.2 (Joseph and Jisha 2009). The culture was incubated at 30 °C and 200 r/min in the dark, and the entire experiment was performed in triplicate. After 7 days, the culture was treated with 2% H_2_O_2_, filtered into a 100-mL volumetric flask mineral powder residues, and then diluted to 100 mL with distilled water. The potassium content in the filtrate was determined by flame photometry (Huang and ChengQun 2010). The data are expressed as mean values with standard deviations.

### Cellulase and hemicellulase activities

Isolates were inoculated into cellulase and hemicellulase fermentation media, respectively, and incubated at 200 r/min and 30 °C for 2–8 days. The cellulase fermentation medium contained (per 1 L): wheat bran, 40 g; peptone, 30 g; (NH_4_)_2_SO_4_, 2.0 g; Na_2_HPO_4_, 0.5 g; MgSO_4_, 0.2 g; FeSO_4_·7H_2_O, 0.1 g; and CaCO_3_, 0.5 g. The hemicellulase fermentation medium contained (per 1 L): wheat bran, 40 g; peptone, 5.0 g; (NH_4_)_2_SO_4_, 3.0 g; KH_2_PO_4_, 5 g; MgSO_4_, 1 g; CaCO_3_, 20 g; NaCl, 0.5 g; KCl, 0.5 g; glucose, 1 g; and Tween 80, 1 mL. Cells were separated from the cultivation medium by centrifugation at 1073×*g* for 10 min at 4 °C, and the supernatants were used as the source of extracellular crude enzyme. Cellulase and hemicellulase activities were determined based on the increase in glucose released from sodium carboxymethyl cellulose and oat xylan, respectively (Miller 1959). One unit of cellulase and hemicellulase activity was defined as the amount of enzyme that produces 1 μmol glucose equivalent per min under the assay conditions. The absorbance was measured using a UV spectrophotometer at 540 nm. The increase in the amount of glucose released was determined using a glucose standard curve. The entire experiment was performed in triplicate, and the data are expressed as mean values with standard deviations.

### Pot experiment design

To assess the effect of the isolates on plant growth, as bio-inoculants, a pot experiment was conducted on wheat using a randomized complete block design with three replicates for each treatment, where with 10 pots per each replicate. Wheat seeds were surface-sterilized with 75% ethanol for 1 min, followed by 1% sodium hypochlorite for 30 min, and extensive wash with sterile water (Collavino et al. 2010). Five sterilized wheat seeds were sown in 10-L clay tile pots containing soil collected from a local field under wheat cultivation. Two treatments were designed (control and CS-1 strain) and performed under natural environmental conditions. For the CS-1 treatment, the roots of 1-week-old plantlets were inoculated with 15 mL of a spore suspension containing fungal spores suspended in sterile water at a density of approximately 10^7^ CFUs/mL. The roots of the control plantlets were inoculated with the same volume of sterile water (Wei et al. 2017). All the pots were irrigated once before sowing to ensure correct seed germination, and then regularly watered during crop growth as per agronomic practices (Reddy and Sudhakara 2014). After 2 months, the wheat seedlings were uprooted separately to determine plant biomass indices. The rhizosphere soil was then carefully collected from 10 random soil cores from pots, and the samples were pooled to yield one composite sample per replicate. The soil samples were passed through a 2-mm sieve, thoroughly homogenized, and stored at − 80 °C for the analysis of microbial community structure.

### PCR amplification and Illumina MiSeq sequencing

To ascertain whether and how the CS-1 strain exerted a positive on the microbial community structure in the wheat rhizosphere, Illumina MiSeq sequencing was used to investigate the differences in microbial communities in the rhizosphere of uninoculated wheat plants and wheat plants inoculated with the CS-1 strain. Total soil genomic DNA was extracted using the Soil DNA kit (USA OMEGA, Bio-Tek, Guangzhou, China), according to the manufacturer’s instructions. The V4–V5 regions of the bacterial *16S rRNA* gene were amplified by using the following primers: 515F (5′-barcode-GTGCCAGCMGCCGCGG-3′) and 907R (5′-CCGTCAATTCMTTTRAGTTT-3′). The ITS rDNA genes from fungal genomic DNA were amplified with the barcoded primers ITS1F (5′-barcode-CTTGGTCATTTAGAGGAAGTAA-3′) and 2043R (5′-GCTGCGTTCTTCATCGATGC-3′). The “barcode” is an eight-base sequence unique to each sample. Amplicons were extracted from 2% agarose gels, purified using the AxyPrep DNA Gel Extraction kit (Axygen Biosciences, Union City, CA, USA), and quantified using QuantiFluor™-ST (Promega, Madison, WI, USA). Purified amplicons were pooled in equimolar amounts and paired-end sequenced (2 × 250) on an Illumina MiSeq platform according to the standard protocols.

#### Processing of Illumina MiSeq sequencing data

Raw fastq files were demultiplexed and quality-filtered using QIIME (http://qiime.sourceforge.net/) with the following criteria. (i) The 300-bp reads were truncated at any site with an average quality score of < 20 over a 50-bp sliding window, discarding the truncated reads shorter than 50 bp. (ii) Reads with exact barcode matching, primer matching with a two-nucleotide mismatch, and reads containing ambiguous characters were removed. (iii) Only sequences with overlaps longer than 10 bp were assembled according to their overlap sequence. Reads that could not be assembled were discarded. Operational taxonomic units (OTUs) were clustered with 97% similarity cut off using UPARSE (version 7.1; http://drive5.com/uparse/), and chimeric sequences were identified and removed using UCHIME (Zheng et al. 2015). The taxonomy of each *16S rRNA* gene sequence was analyzed by RDP Classifier (http://rdp.cme.msu.edu/) against the Silva (SSU123) *16S rRNA* database using a confidence threshold of 70%. The taxonomy of each ITS rDNA gene sequence was analyzed by RDP Classifier against the UNITE 7.0/ITS database (Abarenkov et al. 2010) using a confidence threshold of 70%. To describe community diversity and richness, rarefaction data, Simpson, Shannon, Chao, and ACE indices were created using mothur (http://www.mothur.org/) (Schloss et al. 2009). Raw Illumina sequencing data from the current study were submitted to the NCBI Sequence Read Archive (SRA) under the Accession Number SRP132621.

### Statistical analysis

All experiments were performed in triplicate, and all statistical analyses were performed using SAS version 8.0 software (SAS Institute, Inc.). Differences in mean values were considered significant when *P* < 0.05. The histogram was created using Microsoft Excel 2010 (Microsoft, Redmond, WA, USA).

## Results

### Isolation and screening of phosphofungi

**Fig. 1:**
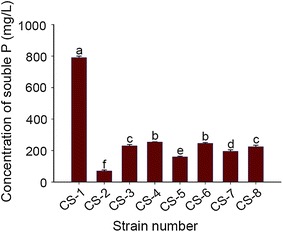
Evaluation of the phosphate solubilising ability of the selected fungi in NBRIP medium. Soluble phosphorus (P) accumulation in cultures of different fungal strains grown on NBRIP medium. Error bars indicate standard errors (n = 3). Columns with different letters are significantly different (*P* < 0.05) according to the Student’s *t*-test

The production of clear zones around colonies on NBRIP medium is an indication that the organism has phosphate-solubilizing ability. In the present study, 20 fungal strains were isolated from a wheat field sampling site, only eight of which (CS-1 to CS-8) showed phosphate-solubilizing capacity. These isolates were further compared by culturing in the NBRIP liquid medium supplemented with Tricalcium phosphate (TCP)(equivalent to 0.1 g phosphorus per 100 mL of medium). As shown in Fig. 1, among these eight strains, CS-1 exhibited the highest phosphate solubilization (790.67 mg/L). The CS-1 strain was therefore selected for experiments determining its other growth-promoting qualities. On the basis of the similar mechanisms of phosphorus and potassium solubilization, the CS-1 strain was also shown to promote efficient potassium release in silicate liquid medium supplemented with feldspar powder. Compared with the control, strain CS-1 led a 127.59% increase in potassium release.

### Identification and phylogenetic analysis of the CS-1 strain

Since strain CS-1 showed the highest phosphate-solubilizing and potassium-dissolving indices among the eight isolates exhibiting phosphate-solubilizing capacity, it was subjected to molecular taxonomy analysis for identification. Based on the phylogenetic analysis of rDNA ITS region sequence, the strain CS-1 was identified as *Aspergillus niger* and has been deposited in the China General Microbiological Culture Collection Center (CGMCC) under the accession numbers CGMCC No. 14634.

### Dynamics of the phosphorus-solubilization mechanism of the CS-1 strain

**Fig. 2:**
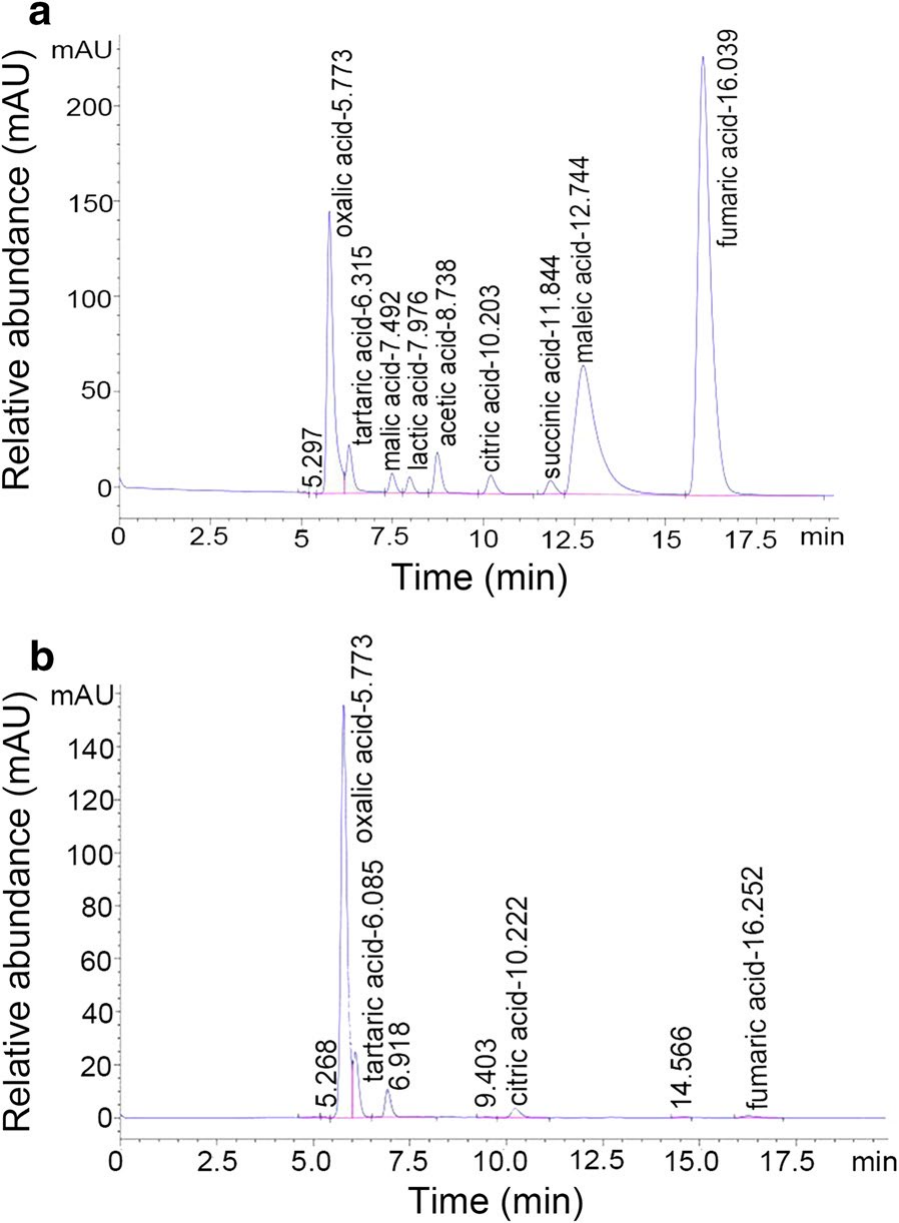
Analyses of organic acids secreted by CS-1 strain were performed by HPLC. HPLC analysis of organic acids. **a** Chromatogram of a standard solution containing the following organic acids: oxalic, tartaric, malic, lactic acid, acetic, citric, succinic, maleic, and fumaric acid. **b** Chromatogram of a 3-day-old culture medium inoculated with strain CS-1, mainly containing oxalic, tartaric, and citric acid

It is generally accepted that phosphate solubilization is linked to a decrease in medium pH. In the current study, the CS-1 strain was inoculated into the NBRIP growth medium to determine the dynamics of its phosphate-solubilization mechanism. The experiment revealed that the pH of the NBRIP medium decreased with an increasing incubation time and that the final pH remained at approximately 2–3 (Table 1), which was consistent with the results presented by Liang et al. (Liang et al. 2011). The biomass of CS-1 strain mycelium increased during the first 3 d of growth, reaching a maximum on the third day, and thereafter, gradually stabilizing before eventually declining (Table 1). Soluble phosphorus content increased to 626 ± 8.50 mg/L in the early growth period, during which it was rapidly solubilized, whereas in the later stages, the dissolved phosphorus content did not significantly increase (Table 1). The HPLC analysis indicated that the mechanism of phosphorus solubilization by strain CS-1 mainly proceeded via the production of oxalic acid (255.74 mg/kg), tartaric acid (201.25 mg/kg), and citric acid (108.04 mg/kg) in the NBRIP medium (Fig. 2).

**Table 1:** Effect of incubation period on the phosphate-solubilizing efficiency of the CS-1 strain

Index	1 day	2 days	3 days	4 days	5 days
pH	3.71 ± 0.02b	3.23 ± 0.41c	2.45 ± 0.26d	2.39 ± 0.11d	2.34 ± 0.07d
Weight_(dry weight/g)_	0.18 ± 0.023d	0.27 ± 0.013c	0.32 ± 0.007a	0.30 ± 0.007ab	0.29 ± 0.004bc
P_(Increment mg/L)_	374.05 ± 53.95b	617.27 ± 34.00a	626.52 ± 8.50a	646.92 ± 5.55a	663.63 ± 22.26a

Values are mean ± SD (n = 3). Means sharing a common letter within the same column are not significantly different at *P *<* 0.05*

### Cellulase and hemicellulase activities

In addition to phosphate-solubilizing and potassium-release abilities, cellulase and hemicellulase activities of the CS-1 strain were also evaluated. The CS-1 strain was, accordingly, cultivated in cellulase and hemicellulase liquid fermentation media and the effects of culture time on the amount of cellulose- and hemicellulose-degrading enzymes produced by CS-1 were determined and compared. As shown in Table 2, the maximal cellulase and hemicellulase activities were 553.39 ± 76.61 and 2979.09 ± 88.68 U/mL after 6 and 3 days of culture, respectively.

**Table 2:** Effect of incubation period on the activity of enzymes produced by the CS-1 strain

Enzyme activity	Incubation time (days)						
	2 days	3 days	4 days	5 days	6 d	7d	8d
Cellulase (U/mL)	1.43 ± 0.56d	124.05 ± 1.15d	159.29 ± 59.96c	375.84 ± 21.17b	553.39 ± 76.61a	345.53 ± 47.68b	347.85 ± 13.57b
Hemicellulase (U/mL)	2743.78 ± 55.97b	2979.09 ± 8888.68a	1655.41 ± 159.54ab	1269.75 ± 53.02c	1251.70 ± 16.34c	997.92 ± 28.90c	_

Values are mean ± SD (n = 3). Means sharing a common letter within the same column are not significantly different at *P *<* 0.05*

### Pot experiment

#### Biomass accumulation

Phosphate-solubilizing bacteria (PSB) have been used as bio-inoculant preparations to improve the growth of plants. On the basis of the aforementioned observations, a pot experiment was conducted to evaluate the practical application of strain CS-1 isolated in the present study. The effect of CS-1 inoculation on plant growth was assessed under natural environmental conditions. Accordingly, strain CS-1 exhibited a stimulatory effect on the growth parameters of wheat seedlings (Table 3), increasing the plant dry biomass (by 23.3%), fresh biomass (by 23.8%), and the root/shoot ratio (31.5%) of wheat, compared with the control group. Although the CS-1 strain did not significantly affect the root length and shoot height, the wheat seedlings treated with this strain had a greater number of lateral roots (Additional file 1: Fig. S1). This would be beneficial for the wheat seedlings, enabling them to obtaining sufficient water and nutrients from the soil.

**Table 3:** Effect of the CS-1 strain on growth parameters of wheat seedling

Treatment	Shoot height (cm)	Root length (cm)	Dry weight (g)	Fresh weight (g)	Root/shoot ratio
CK	40.76 ± 0.83a	17.78 ± 1.16a	0.30 ± 0.04b	1.26 ± 0.16b	0.1809 ± 0.011b
CS-1	40.53 ± 1.20a	18.15 ± 1.77a	0.37 ± 0.05a	1.56 ± 0.06a	0.2378 ± 0.011a

Values are mean ± SD (n = 30). Means sharing a common letter within the same column are not significantly different at *P *<* 0.05*. “CS-1” denotes wheat seedlings treated with a suspension of strain CS-1 spores. “CK” denotes wheat seedlings treated with an equal volume of sterile water

#### Illumina MiSeq sequencing and sequence analysis

**Fig. 3:**
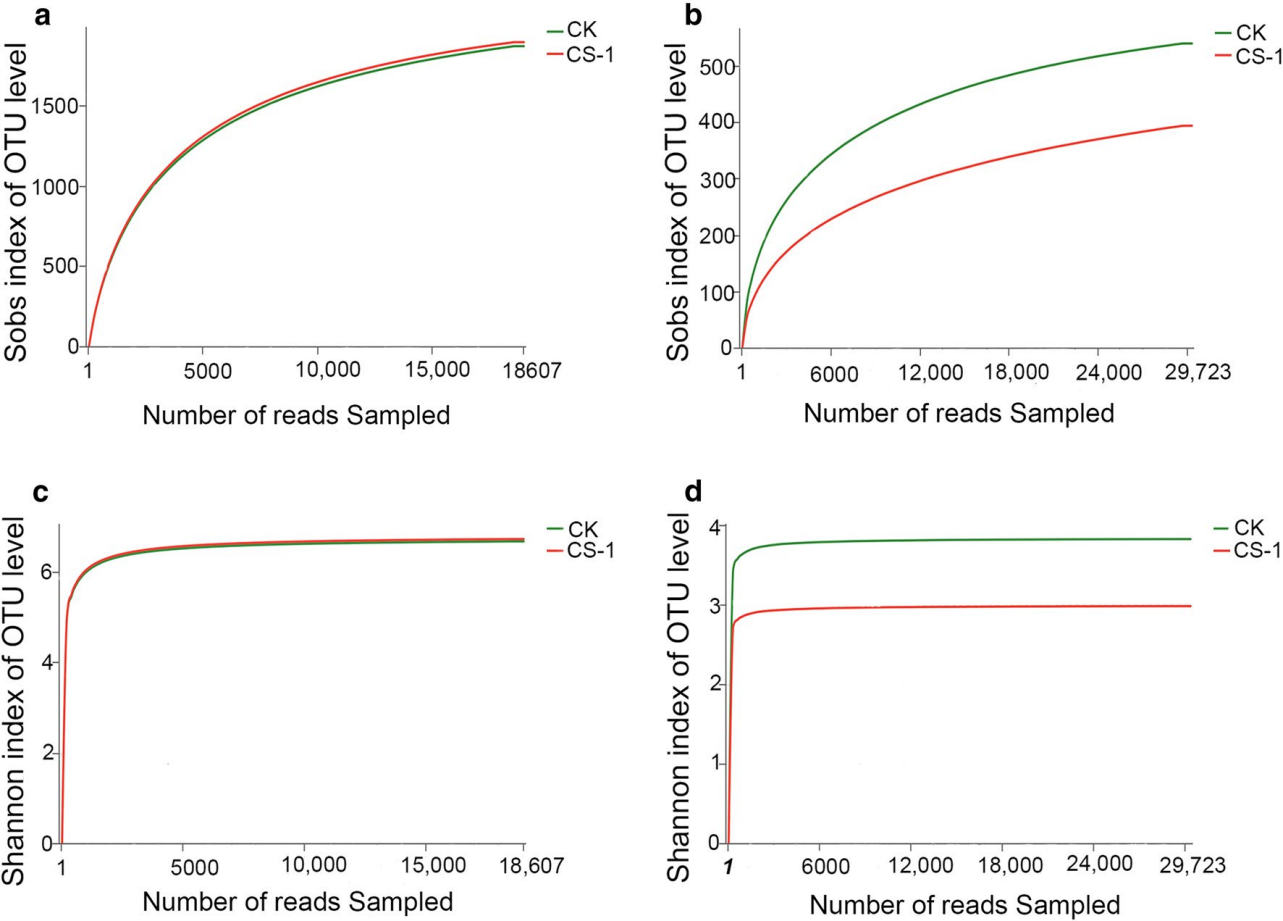
Rarefaction and Shannon curve of CS-1 and control treatments. Fungal and bacterial rarefaction curves and Shannon curves depicting the effect of CS-1 and control treatments on the number of OTUs. **a** Rarefaction curves of bacteria from CS-1 and control treatments. **b** Rarefaction curves of fungi from CS-1 and control treatments. **c** Shannon curves of bacteria from CS-1 and control treatments. **d** Shannon curves of fungi from CS-1 and control treatments

More than 20,000 bacterial and 30,000 fungal valid reads were obtained for each replicate a sequence optimization process. Using a 3% dissimilarity cut-off for clustering, the reads were grouped into different OTUs (Table 4). Rarefaction curves (Fig. 3a, b) for the bacterial and fungal communities at distance levels of 0.03 did not fully represent the number of different bacterial and fungal communities, while the Shannon diversity curves approached a plateau with the increase in sequencing number (Fig. 3c, d). Furthermore, the coverage was approximately > 99%. Therefore, the sequencing capability was sufficiently large to capture the complete diversity of these communities. The Chao and ACE values are indicators of species richness, and Shannon and Simpson indices are indicators of species diversity (Liu et al. 2015). The bacterial community richness and diversity indices were calculated (Table 4); no significant differences observed between the two soil samples. The fungal community richness index is also presented in Table 4. The higher ACE and Chao values in the control samples indicated that the CS-1 strain treatment maintained a lower richness within the fungal community. The Simpson index and Shannon diversity index values showed a similar trend for fungal diversity.

**Table 4:** Diversity and richness indices of bacterial and fungal community from CS-1 treatment and control

Index	Bacteria		Fungi	
	CK	CS-1	CK	CS-1
OTU	1895.33 ± 38.66a	1868.33 ± 7.54a	539.67 ± 7.13a	393.67 ± 49.57b
Shannon	4.39 ± 0.52a	4.46 ± 0.09a	3.83 ± 0.17a	2.98 ± 0.48b
Simpson	0.05 ± 0.04a	0.03 ± 0.01a	0.062 ± 0.02a	0.14 ± 0.02a
Ace	412.75 ± 9.22a	406.65 ± 9.35a	611.01 ± 14.97a	502.11 ± 26.11b
Chao	414.42 ± 5.71a	417.76 ± 17.06a	588.30 ± 43.30a	478.36 ± 12.77b
Coverage	0.9978	0.9977	0.9966	0.9963

Chao and ACE value are indicators of species richness. Shannon and Simpson are indicators of species diversity. Values are mean ± SD (n = 3). Means sharing a common letter within the same column are not significantly different at *P *<* 0.05*

#### Comparison of bacterial communities in the CS-1 and control

**Fig. 4:**
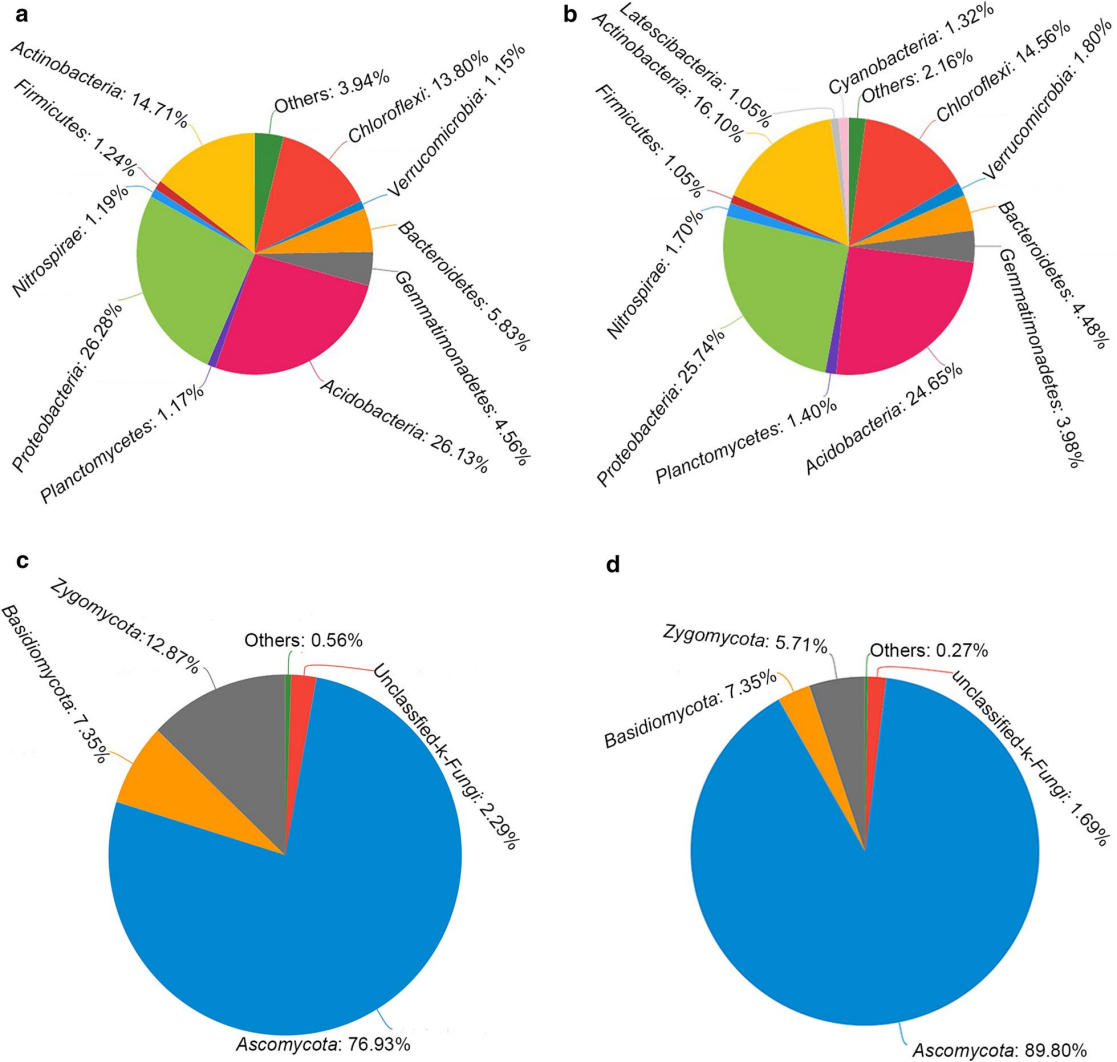
Relative abundance of the dominant bacterial and fungal phyla in CS-1 and control treatment. The relative abundance (%) of all bacteria and fungi on the phylum level in the rhizosphere soil of CS-1 and control treatments. **a** The relative abundance of all detected bacterial phyla after the control treatment. **b** The relative abundance of all detected bacterial phyla after the CS-1 treatment. **c** The relative abundance of all detected fungal phyla control treatment. **d** The relative abundance of all detected fungal phyla after the CS-1 treatment

**Fig. 5:**
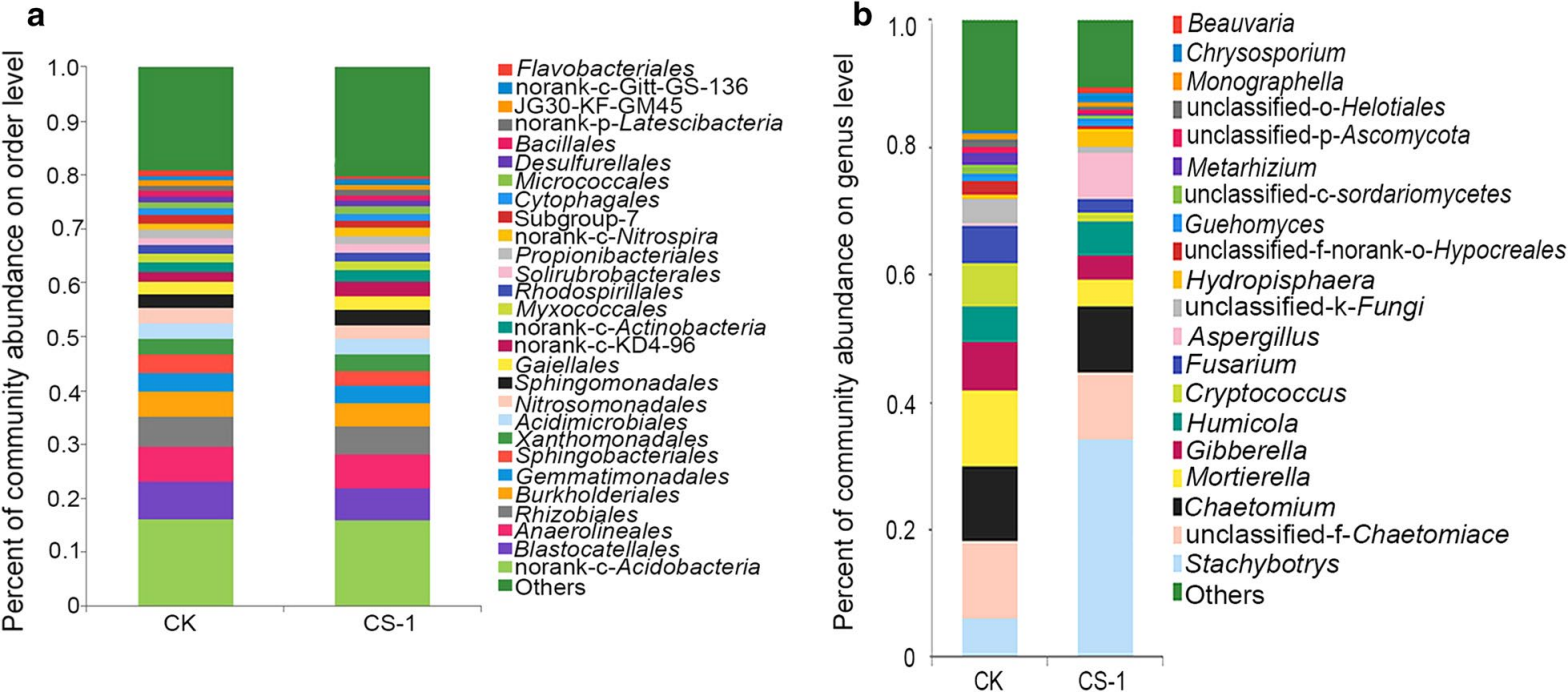
Relative abundance of bacteria (genus levels) and fungi (order levels) in CS-1 and control treatments. The relative abundance (%) of all bacteria (**a**) and fungi (**b**) on the order and genus levels, respectively, in the rhizosphere soil of CS-1 and control treatments

All bacterial sequences were classified at the phylum level down to the genus level using the Mothur program, and the relative abundances of the assigned phyla and genera were compared between the two samples. The overall bacterial composition and the distribution of each group in the different treatments were similar. At the phylum level, 11 and 13 bacteria phyla were detected in the control group and CS-1 treatment group, respectively (Fig. 4a, b), with *Proteobacteria*, *Acidobacteria*, *Actinobacteria*, and *Chloroflexi* the top four bacterial phyla. The average abundances of these phyla in the two treatment groups were 26.28, 26.13, 14.71 and 13.80%, respectively, in the control; and 25.74, 24.65, 16.10 and 14.56%, respectively, in the CS-1 treatment group. At the order level, the bacterial composition and the relative abundance of the assigned groups also did not differ in the samples (Fig. 5a).

#### Differences in fungal diversity between CS-1 treatment and control samples

**Fig. 6:**
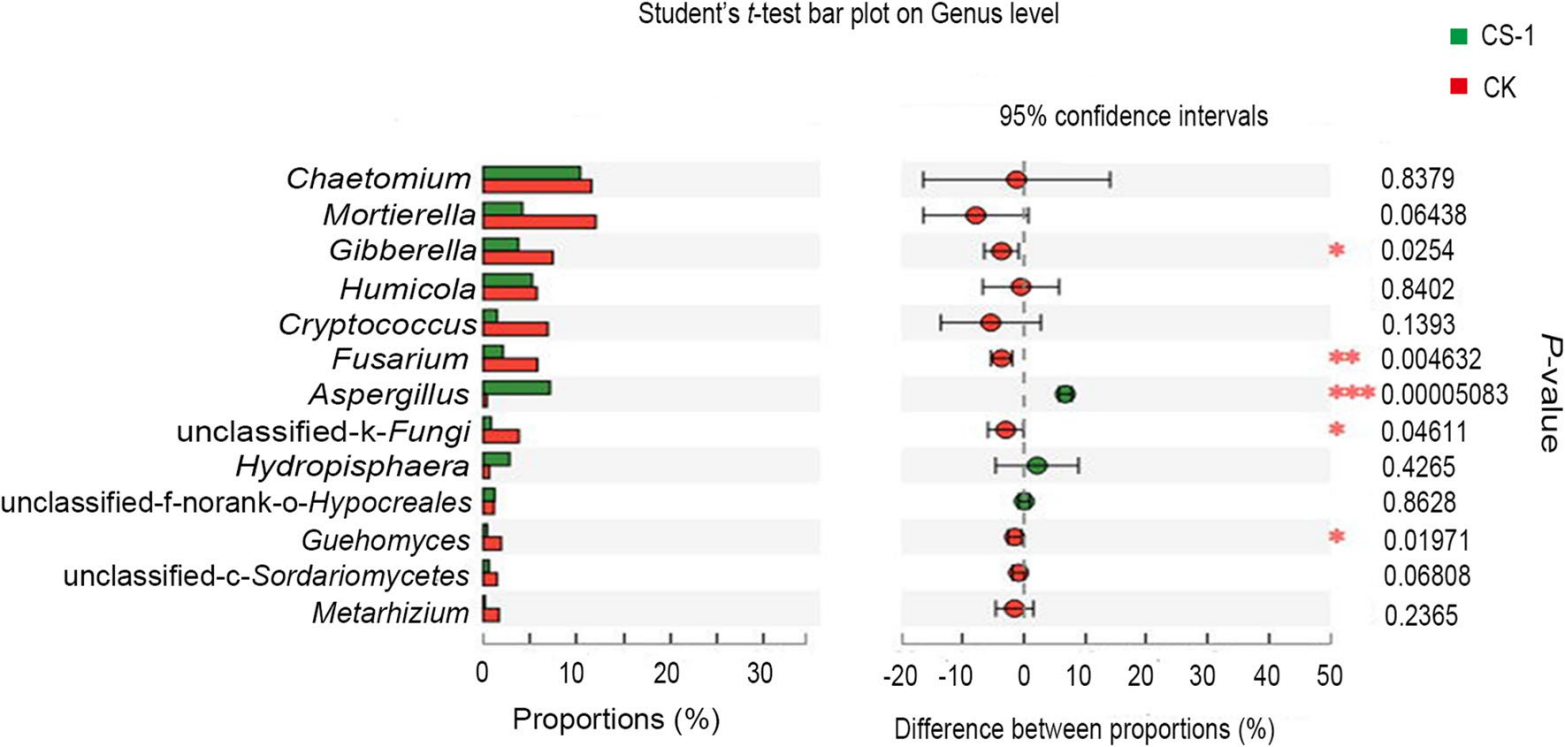
Multi pathogenic fungal genera difference test between CS-1 and control treatments. Multi-pathogenic fungal genera difference test between CS-1 treatment and control treatments. Error bars indicate standard errors (n = 3). Columns with different letters are significantly different (*P *< 0.05) according to Student’s *t*-test

All fungal sequences were classified at the phylum level down to the genus level using the Mothur program. Three known fungal phyla were detected, with *Ascomycota* representing the most dominant phylum and accounting for 76.93 and 89.80% in the control groups and CS-1 treatments respectively (Fig. 4c, d). We found that the overall fungal composition in the different treatment groups was similar, while the distribution of each phylum or group varied (Fig. 5b). The surprising finding was that the content of *Gibberella* and *Fusarium*, which cause soil-borne diseases in wheat and other crops, were significantly lower in treatment than in the control, while the *Aspergillus* contents were significantly higher (Fig. 6). Furthermore, after mining useful information hidden in the original data (Additional file 1: Table S1), the relative abundances of *Monographella*, *Bipolaris*, and *Volutella* in the CS-1 treatment group were found to decrease.

## Discussion

Phosphate is a nutrient that is limiting for the growth of crops, with only 0.1% of total phosphorous in the soil available to plants (Vessey and Heisinger 2001). Although in China, the soil may harbor a total phosphorus reserve, it cannot be efficiently used by the plants because of low soil quality (Wu 2004). PSMs able to release phosphorus from the soil minerals play a key role in soil fertility (Wakelin et al. 2012) when P availability is low or P demand is high. Most relevant literature pertains to PSB and their potential use for the enhancement of soil fertility (Collavino et al. 2010; Ghosh et al. 2012). However, a few filamentous nonmycorrhizal fungi are also involved in phosphate solubilization, especially *Aspergillus* spp. and *Penicillium* spp. (Altomare et al. 1999; Singh 2011). The beneficial effects for various crops of such phosphate-solubilizing fungi (PSF) as *Aspergillus* (Mittal et al. 2008), *Trichoderma* (Zayed and Abdelmotaal 2005), *Penicillium* (Reyes et al. 2002), and vesicular arbuscular mycorrhizae (Omar 1997) have been demonstrated.

In the present study, the formation of clear zones around the colonies on NBRIP medium indicated the phosphate-solubilizing ability of strain, with the extent of phosphate-solubilization determined quantitatively by liquid fermentation methods. Eight PSF strains were isolated on the NBRIP medium (Fig. 1). The results indicated that the NBRIP medium combined with the Martin agar medium supplemented with 30 μg/mL streptomycin constituted an effective method for isolating and screening of PSF from the rhizosphere soil samples. We report here the isolation of *A. niger* CS-1, which exhibited the highest phosphate-solubilizing ability among the eight fungal isolates tested. It is generally accepted that the major mechanism of mineral phosphate solubilization involves the action of organic acids synthesized by PSMs (Chabot et al. 1993; Morales et al. 2007). In the current study, the types and quantities of the organic acids secreted by strain CS-1 were analyzed by HPLC (Fig. 2). The major organic acids secreted by this strain were oxalic, tartaric, and citric acids, which was consistent with a previous report by Li et al. (2016). However, Tkacz and Lange demonstrated that the main organic acids produced by *A. niger* are gluconate, citrate, and oxalate (Tkacz and Lange 2004). This discrepancy may be associated with the different strain sources. Although the strains were identified as *A. niger*, they may exhibit some differences in the biosynthetic pathways and biological activities (Mäkelä et al. 2002; Yin et al. 2015). Moreover, the type of organic acids secreted by *A. niger* is also affected by the medium type and culture conditions (Altomare et al. 1999).

Enormous amounts of agricultural cellulosic wastes are accumulating because of the low efficiency and high cost of their utilization (Lee et al. 2008). It has been reported that fungi are potent cellulase and hemicellulase producers, and that, among fungal species, *Aspergillus* spp. notably produces copious amounts of such enzymes (Li et al. 2014, 2015b; Pourramezan et al. 2012). In the current study, the CS-1 strain exhibited a better cellulose-degrading activity (Table 2) than other strains (Li et al. 2003; Sachslehner et al. 1997; Pourramezan et al. 2012). It is worth noting that the carbon source in the growth medium considerably affects the synthesis of cellulolytic enzymes in liquid culture, with the highest amounts of cellulases produced by *A. niger* with sodium carboxymethylcellulose as a carbon source (Gautam et al. 2010).

An important quality for the potential use of PSMs growth-stimulating ability upon when plants are inoculation (Collavino et al. 2010). Accordingly, the strain *A. niger* CS-1 promoted plant growth by increasing the fresh and dry mass of wheat per plant in pot experiments. It is generally accepted that PSF solubilize elemental phosphorus from insoluble RP for plant growth. To reveal the mechanism underpinning the plant growth-promoting effect of the CS-1 strain, Illumina MiSeq sequencing was used to characterize the soil microorganism communities after control and CS-1 treatments. Understanding the ecology of these taxa is important as it may provide new insights about soil biological resources to foster sustainable agricultural production (Adesemoye and Kloepper 2009; Wakelin et al. 2012).

*Proteobacteria*, *Actinobacteria*, *Acidobacteria*, and *Chloroflexi* were the four most dominant phyla in the control and CS-1–treated samples. The *Proteobacteria* phylum includes many of the bacteria responsible for nitrogen fixation (Chen et al. 2003; Wang et al. 2015) and a wide variety of pathogens (Tsolis 2002). *Actinobacteria* play an important role in the decomposition and humus formation processes (Kopecky et al. 2011). The ecology and metabolism of *Acidobacteria* is not well understood because the majority of these bacteria have not been cultured (Sun et al. 2014). *Chloroflexi* constitutes a specialized group of filamentous bacteria that are only active under aerobic conditions, consume primarily carbohydrates, and contribute to the overall filament index (Kragelund et al. 2007). Nevertheless, no significant differences were apparent between the two treatment treatments analysis on the phylum level or the order level. Surprisingly, the experiment revealed that the inoculation with the CS-1 strain reduced the diversity of the soil fungal communities (Table 4), although the overall fungal community composition was largely unaffected. The differences between the control and CS-1 treatments were reflected in the distribution of each fungal group, including uncultured species. This indicated that the impact of the CS-1 strain on the microbial community in the wheat rhizosphere was complex, and particularly, led to a significant reduction of the content of pathogenic fungi. It is worth noting that the CS-1 strain did not hinder the growth of plant pathogens (such as *Fusarium oxysporum*, *Bipolaris sorokiniana*, and *Gibberella fujikuroi*) during dual-plate confrontation assays on potato dextrose agar plates (There’s no data show). Hence, the CS-1 inoculation effect may be associated with an ecological niche competition, because the relative abundance of *Aspergillus* spp. increased significantly in the CS-1 treatment group, whereas these fungi were undetected in the control group. To the best of our knowledge, this is the first-ever report demonstrating the effect of *A. niger* on the microbial community structure.

In conclusion, in the current study, we isolated an efficient phosphofungus, CS-1, which was subsequently identified as *A. niger.* This is the first study to comprehensively elaborate the mechanisms of *A. niger* activity as a plant growth promoting rhizobacteria (PGPR), including phosphorus and potassium solubilization, cellulase and hemicellulase activity, and inhibition of pathogenic fungi in the crop rhizosphere soil. Further, the CS-1 strain exhibited marked growth-promoting effects on wheat seedlings. Owing to its multiple beneficial effects, the *A. niger* CS-1 strain has the potential to be developed and commercially formulated as a commercial microbial agent for field application.

## Additional file

**Additional file 1: Fig. S1.** Effect of the CS-1 strain on root length and shoot height of wheat seedings. As compared with control, wheat seedlings treated with this strain had a greater number of tillers and lateral roots. **Table S1.** Percent of pathogenic fungi abundance.

## Abbreviations

RP: rock phosphate
PSMs: phosphate-solubilizing microorganisms
NBRIP: National Botanical Research Institute’s phosphate
ITS: internal transcribed spacer
PSF: phosphate-solubilizing fungal
HPLC: high-performance liquid chromatography
TCP: tricalcium phosphate
PSB: phosphate-solubilizing bacteria
PGPR: plant growth promoting rhizobacteria
BLAST: Basic Local Alignment Search Tool
