# Supplementary material for: Isolation and characterization of phosphofungi, and screening of their plant growth-promoting activities
Source: AMB Express. 2018 Apr 20;8:63. doi: 10.1186/s13568-018-0593-4 (PMC5910442; doi:10.1186/s13568-018-0593-4)
Supplement: Supplementary file 1 — Additional file 1: Fig. S1. Effect of the CS-1 strain on root length and shoot height of wheat seedings. As compared with control, wheat seedlings treated with this strain had a greater number of tillers and lateral roots. Table S1. Percent of pathogenic fungi abundance. [file 13568_2018_593_MOESM1_ESM.pdf]

Additional file

Isolation and characterization of phosphofungi and screening for plant growth-promoting activities

AMB Express

Xiaohui Wang[1], Changdong Wang[2], Junkang Sui[1], Zhaoyang Liu[2], Qian Li[2], Chao Ji[2], Xin Song[2], Yurong Hu[2], Changqian Wang[2], Rongbo Sa[1], Jiamiao Zhang[2], Jianfeng Du[2], Xunli Liu[2]*

1 College of Life Science, Shandong Agriculture University, No. 61, Daizong Street, Taian, Shandong 271018, China

2 College of Forestry, Shandong Agricultural Universities, No. 61, Daizong Street, Taian, Shandong 271018, China

* Author for correspondence, E-mail: xunliliu@163.com

Tel: 0086 0538 8249131

Fax: 0086 0538 8249164

Figure S1

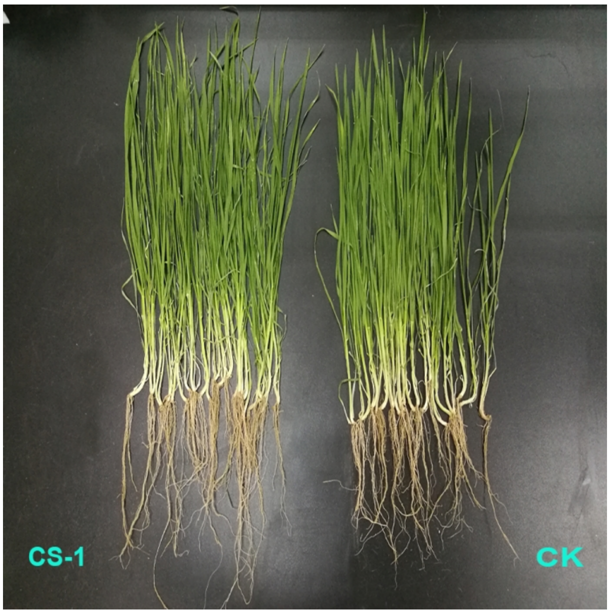

Fig.S1 Effect of the CS-1 strain on root length and shoot height of wheat seedings.As compared with control, wheat seedlings treated with this strain had a greater number of tillers and lateral roots.

Table S1 Percent of pathogenic fungi abundance

Treatment	Fusarium	Gibberella	Volutella	Bipolaris	Monographella
CK	5.83%±0.00a	7.45%±0.01a	0.73%±0.00a	0.02%±0.00a	1.13%±0.00a
CS-1	2.15%±0.00b	3.79%±0.01b	0.10%±0.00a	0.01%±0.00a	0.50%±0.00a

Notes: Values are the means ± SD (n = 3). Means sharing a common letter within the same column are not significantly different at $P < 0.05$.
